# Sick Building Syndrome by Indoor Air Pollution in Dalian, China

**DOI:** 10.3390/ijerph10041489

**Published:** 2013-04-11

**Authors:** Peng Guo, Kazuhito Yokoyama, Fengyuan Piao, Kiyoshi Sakai, Md Khalequzzaman, Michihiro Kamijima, Tamie Nakajima, Fumihiko Kitamura

**Affiliations:** 1 Department of Epidemiology and Environmental Health, Juntendo University Faculty of Medicine, Bunkyo-ku, Tokyo 113-8421, Japan; E-Mails: kakuchina_1@yahoo.co.jp (P.G.); fkitamu@juntendo.ac.jp (F.K.); 2 Faculty of Environmental and Information Science, Yokkaichi University, 1200 Kayouchyou, Yokkaichi, Mie 512-8512, Japan; 3 Department of Public Health and Occupational Medicine, Mie University Graduate School of Medicine, Tsu, Mie 514-8507, Japan; 4 Department of Occupational and Environmental Health, Dalian Medical University, Dalian, LiaoNing 116044, China; E-Mail: piaofengjp@yahoo.co.jp; 5 Nagoya City Public Health Research Institute, Mizuho-ku, Nagoya, Aichi 467-8615, Japan; E-Mail: ehdeiken@sa.starcat.ne.jp; 6 Department of Occupational and Environmental Health, Nagoya University Graduate School of Medicine, Showa-ku, Nagoya, Aichi 466-8550, Japan; E-Mails: romenraihan@yahoo.com (M.K.); tnasu23@med.nagoya-u.ac.jp (T.N.); 7 Department of Occupational and Environmental Health, Nagoya City University Graduate School of Medical Sciences, Mizuho-ku, Nagoya, Aichi 467-8601, Japan; E-Mail: kamijima@med.nagoya-cu.ac.jp

**Keywords:** sick building syndrome, indoor air pollution, China, formaldehyde, volatile organic compound

## Abstract

This study assessed subjective symptoms related to indoor concentrations of chemicals among residents in a housing estate in Dalian, China, where indoor air pollution by interior decoration materials has recently become a major health problem. Fifty-nine males and 50 females were surveyed for their symptoms related to sick building syndrome. Formaldehyde (HCHO), NO_2_, and volatile organic compounds (VOCs) in their dwellings were collected using a diffusion sampler and measured by GC/MS. For residents with one or more symptoms in the past, HCHO, butanol or 1,2-dichloroethane concentrations were significantly greater in their bedrooms or kitchens compared with those of subjects without previous symptoms. For residents with one or more symptoms at the time of the study, 1,1,1-trichloroethane, xylene, butanol, methyl isobutyl ketone, and styrene concentrations in their bedrooms or kitchens were significantly greater compared with those of residents without symptoms. HCHO, NO_2_, and VOCs were detected in all rooms, but their levels were lower than the guideline values except for HCHO in two rooms. Chemical substances from interior decoration materials at indoor air levels lower than their guideline values might have affected the health status of residents.

## 1. Introduction

Sick building syndrome (SBS) is a medical condition in which people in a building suffer from symptoms of illness or feeling unwell for no apparent reasons [1]. The symptoms tend to increase in severity as a function of time spent in the building and improve over time or even disappear when people are away from the building [1]. A variety of factors in the home environment are related to SBS [2,3,4]. Indoor volatile organic compounds (VOCs) and their relationship to SBS were demonstrated in studies at nonmanufacturing workplaces [3,5,6]. In addition, SBS was observed at indoor ambient air levels greater than 21.3, 15.5, 4.40, 3.90, 4.60, 3.80, 4.60 and 8.20 μg/m^3^ for formaldehyde (HCHO), NO_2_, butyl acetate, ethylbenzene, α-pinene, *p*-dichlorobenzene, nonanal and xylene, respectively [2,6,7]. However, for butanol, methyl isobutyl ketone, styrene, and toluene, no symptoms were reported at 4.20–190.2, 3.80–44.1, 4.70–171.3 and 3.00–3,104.7 μg/m^3^, respectively [2].

The home indoor environment is considered an important factor affecting health [2,4,8,9,10,11,12,13]. In recent years, indoor air pollution by interior decoration materials has become a major health problem in China; symptoms related to the nervous system or respiratory organs among inhabitants in Wuhan, Beijing, Ningbo and Taiyuan have been widely reported [7,10,11,12,13]. Indoor air pollution, thus, has become a strong public concern in China. The present study reports subjective symptoms in the past and at the time of study, in relation to indoor concentrations of HCHO, VOCs, and NO_2_ at the time of study. The health effects among residents in the dwellings with low levels of exposures were investigated.

## 2. Experimental Section

### 2.1. Subjects and Their Houses

The study was conducted during from 5 to 22 August 2007, in Dalian, China. The area surveyed was a housing estate consisting of about 3,000 houses located approximately 10 km north from the center of Dalian city. Through the local council committee, volunteers were invited to participate in the survey. Just prior to the study, a meeting was held to explain to participants the purpose of the study, and representatives from approximately 100 families attended. Seventy families agreed to participate in the study; questionnaires were distributed to collect data about their demographic characteristics and subjective symptoms in the past (since they moved into their flats) and at the time of study. Finally, completed questionnaires were collected by 109 residents (59 males and 50 females) of 59 households. Among them, 1 female and 23 males were smokers. The ages for the males and females were 23–77 and 25–75 years, respectively. The participants had lived in their flats for 5–154 months (mean 45). They were at home for 13 h per day on average during the study. The participants were not exposed to toxic substances, such as organic solvents and heavy metals, at the workplace.

The surveyed residents lived in nine reinforced-concrete buildings. Each house had an area of 40.0–158.0 m^2^ (80.0 on average) with one bedroom, living room, and kitchen. Residents reported that water-based paint and solvent-based paint were used in 33 and five houses, respectively; no answers were obtained from 21 households. A total number of five windows were in each house.

### 2.2. Sampling Methods

HCHO and NO_2_ were collected using a diffusion sampler packed with silica gel containing triethanolamine (passive gas tube for HCHO and NO_2_, Sibata Scientific Technology, Tokyo, Japan). VOCs were collected using a diffusion sampler packed with activated charcoal (passive gas tube for organic solvents, Shibata Scientific Technology). The sampling time was approximately 24 h. Sampling was performed in the center of the bedroom and kitchen of each flat at a height of 1.2–1.5 m above the floor [4,14]. The windows were open for 10 h on average during the sampling. The average temperature was 28 °C in the bedrooms and kitchens. To measure outdoor air concentrations of chemicals, diffusion samplers were hung with strings from the window frames of houses (39 samplers, 2–3 per building).

### 2.3. Analytical Methods

The samplers were stored at −4°C and then transported on ice by air to Japan. The analysis was conducted at the Nagoya City Public Health Research Institute. HCHO and NO_2_ were extracted with distilled water and analyzed by spectrophotometry by the 4-amino-3-hydrazino-5-mercapto-1,2,4-triazole method and sulfanilamide method, respectively. VOCs were extracted with carbon disulfide and analyzed with a gas chromatograph equipped with a mass spectrometer (GC/MS). The GC/MS was equipped with a 60 × 0.25 mm i.d. capillary column coated with a 1.5-µm film (NB-1, GL Sciences, Tokyo, Japan). The GC oven temperature was first maintained at 45 °C for 5 min, then programmed to 300 °C at 10 °C/min and held at 300 °C for 7 min. The analysis was performed with a helium flow rate of 0.9 mL/min generally under a selected ion monitoring mode targeting chemicals [4]. The analyzed substances and their exposure limits are listed in Table 1 [15].

**Table 1:** Indoor exposure limits (μg/m^3^) of WHO , China and Japan [15].

	WHO	China	Japan
Formaldehyde (HCHO)	100	100	100
VOCs (total)	-	600	400
Benzene (C_6_H_6_)	4.4–7.5 × 10^−^^6^	110	-
Butanol (C_4_H_10_O)	-	-	-
Butyl acetate (C_6_H_12_O_2_)	-	-	-
Carbon tetrachloride (CCl_4_)	-	-	-
Chloroform (CHC_l3_)	4.2 × 10^−^^7^	-	-
1,2-Dichloroethane (C_2_H_4_C_l2_)	-	-	-
*p*-Dichlorobenzene (C_6_ H_4_C_l2_)	-	-	240
Ethyl acetate (C_4_H_8_O_2_)	-	-	-
Hexane (C_6_H_14_)	-	-	-
Methyl ethyl ketone (CH_3_COC_2_H_5_)	-	-	-
Methyl isobutyl ketone (CH_3_COCH_2_CH(CH_3_)_2_)	-	-	-
Styrene (C_6_H_5_C_2_H_3_)	260	-	220
1,1,1-Trichloroethane (CH_3_CCl_3_)	-	-	-
Tetrachloroethylene (C_2_Cl_4_)	250	-	-
Trichloroethylene (C_2_HCl_3_)	4.3 × 10^−^^7^	-	-
Toluene (C_6_H_5_CH_3_)	260	200	260
*o*-Xylene (C_8_H_10_)	-	-	-
*p*-Xylene (C_8_H_10_)	-	-	-
Total xylenes (C_8_H_10_)	870	200	870
NO_2_	200	240	-

‒: no exposure limit values.

### 2.4. Questionnaire

The self-administered questionnaire used in the present study was derived from one that we used in our previous study on sick building symptoms [16]. The set of questions was originally developed to investigate subjective symptoms of workers exposed to organic solvents [17].

### 2.5. Statistical Methods

The mean concentrations of HCHO, NO_2_, and VOCs were calculated as geometric means because they were approximately log normally distributed. When concentrations were below the detection limit, they were set at half of the detection limit in calculating the geometric mean. Differences in air concentrations of chemicals in rooms between residents with and without experience of symptoms were examined by Wilcoxon’s rank sum test. These calculations were performed with SPSS for Windows, version 14.0 software.

Point estimation and 95% confidence intervals of the median of measured values were calculated using an open-access JAVA script program [18] based on the method described by Altman *et al.* [19].

### 2.6. Ethical Issues

The research protocol was reviewed by the Research Ethics Committee of Nagoya University, and the study was performed under their approval (No. 552, 3 August 2007).

## 3. Results and Discussion

Indoor air concentrations of HCHO, VOCs, and NO_2_ in 59 houses are shown in Table 2. Among substances with exposure limit values (Table 1), HCHO concentrations exceeded the exposure limit (100 μg/m^3^) in one bedroom (272 μg/m^3^) and in one kitchen (167 μg/m^3^). The number and percentage of the values under the exposure limits are listed in Appendix 1. Point estimation and 95% confidence intervals of median concentrations are shown in Appendix 2. None of other substances with exposure limits (Table 1) showed indoor air concentrations greater than the exposure limits. Reasons for high HCHO levels observed could not be elucidated in this study.

**Table 2:** Indoor concentrations ^a^ of HCHO, VOCs, and NO_2_ in 59 houses in Dalian, China (μg/m^3^, 5–23 August 2007).

	Bedroom ^b^		Kitchen ^b^		Outdoor ^c^		Detection limit
	Median ^d^	Range	Median ^d^	Range	Median ^d^	Range	
Formaldehyde (HCHO)	29	13–272	30.6	13–167	14	<DL–40	15
VOC (total)	67.9	36.5–202	66.3	37.3–260	54.5	35.0–101	5.6
Benzene (C_6_H_6_)	2.4	<DL–15.7	2.8	<DL–32.4	2.5	<DL–5.5	1.0
Butanol (C_4_H_10_O)	9.8	<DL–65.2	8.6	<DL–61.1	5.3	<DL–10.5	4.4
Butyl acetate (C_6_H_12_O_2_)	3.7	<DL–27.3	3.6	<DL–31.7	3.07	<DL–12.4	1.1
Carbon tetrachloride (CCl_4_)	0.7	<DL–1.8	0.9	<DL–11.9	0.7	<DL–1.8	0.5
Chloroform (CHC_l3_)	<DL	<DL–2.3	<DL	<DL–2.2	<DL	<DL–1.8	1.6
1,2-Dichloroethane (C_2_H_4_C_l2_)	1.0	<DL–65.4	1.1	<DL–11.3	1.0	<DL–3.1	0.5
*p*-Dichlorobenzene	<DL	<DL–111	<DL	<DL–59.0	<DL	<DL–8.5	0.5
(C_6_ H_4_C_l2_)							
Ethyl acetate (C_4_H_8_O_2_)	<DL	<DL–<DL	<DL	<DL–<DL	<DL	<DL–<DL	41
Hexane (C_6_H_14_)	1.5	<DL–6.3	1.59	<DL–18.1	1.6	<DL–4.0	0.4
Methyl ethyl ketone	<DL	<DL–3.3	<DL	<DL–3.3	<DL	<DL–6.5	1.2
(CH_3_COC_2_H_5_)							
Methyl isobutyl ketone	<DL	<DL–1.4	<DL	<DL–1.1	<DL	<DL–0.8	0.4
(CH_3_COCH_2_CH(CH_3_)_2_)							
Styrene (C_6_H_5_C_2_H_3_)	<DL	<DL–2.3	<DL	<DL–2.8	<DL	<DL–1.7	0.8
1,1,1-Trichloroethane (CH_3_CCl_3_)	0.6	<DL–3.0	0.6	<DL–1.1	0.6	<DL–1.1	0.4
Tetrachloroethylene (C_2_Cl_4_)	<DL	<DL–10.0	<DL	<DL–16.1	<DL	<DL–22.3	0.4
Trichloroethylene (C_2_HCl_3_)	<DL	<DL–1.7	<DL	<DL–<DL	<DL	<DL–<DL	0.9
Toluene (C_6_H_5_CH_3_)	14.1	2.4–97.4	12.0	3.0–80.0	10.8	3.9–40.6	0.5
*o*-Xylene (C_8_H_10_)	1.8	<DL–9.6	1.7	<DL–39.7	1.4	<DL–5.1	0.5
*p*-Xylene (C_8_H_10_)	5.7	0.7–23.6	6.5	1.0–111	5.4	<DL–17.0	0.7
Total xylenes (C_8_H_10_)	7.3	<DL–33.3	8.3	1.2–151	6.9	<DL–22.1	1.2
NO_2_	20.5	<DL–72.1	40.0	<DL–205	27.1	<DL–78.2	5.6

^a^ <DL = Lower than detection limits; number of samples below the detection limit are listed in Appendix 1. ^b^ Each house had one bedroom and kitchen, *i.e.*, 59 samples were taken from each house. ^c^ A total of 39 samples. ^d^ Point estimation and confidence intervals are shown in Appendix 2.

Geometric mean, geometric standard deviation after log transformation, and 5th and 95th percentiles of HCHO, VOCs, and NO_2_ concentrations are presented in Table 3. For chemicals with exposure limit values in Table 1, their 95th percentile values were less than the exposure limit. Therefore, residents were exposed to very low level of chemicals at the time of study.

**Table 3:** Geometric mean concentrations (GM), geometric standard deviation (GSD) and 5th and 95th percentiles of HCHO, VOCs, and NO_2_ inside the bedroom and kitchen in the sampled flats (calculated from Table 2).

	Bedroom				Kitchen			
	GM	GSD	5th percentile	95th percentile	GM	GSD	5th percentile	95th percentile
HCHO	33.5	1.73	16	82	32.3	1.70	14.9	91.0
VOCs (total)	74.1	1.56	37.5	187	73.5	1.45	43.3	154
Benzene	2.54	1.78	1.1	8.7	2.89	1.86	1.1	8.6
Butanol	9.03	2.06	2.2	34	8.62	1.77	2.2	20.9
Butyl acetate	3.69	2.14	1.2	16.4	3.51	1.98	1.1	11.3
Carbon tetrachloride	0.64	2.00	0.2	1.6	0.72	2.09	0.2	1.4
Chloroform	0.89	1.28	0.8	2.2	0.86	1.23	0.8	2.0
1,2-Dichloroethane	1.38	2.97	0.3	17.9	1.29	2.11	0.6	5.9
*p*-Dichlorobenzene	0.49	3.89	0.3	17.7	0.46	3.16	20.3	8.6
Hexane	1.43	1.97	0.2	4.0	1.61	2.14	0.2	8.0
Methyl ethyl ketone	0.80	1.63	0.6	2.3	0.79	1.67	0.6	2.5
Methyl isobutyl ketone	0.35	1.87	0.2	0.9	0.33	1.88	0.2	0.9
Styrene	0.57	1.83	0.4	1.8	0.61	2.0	0.4	2.2
1,1,1-Trichloroethane	0.52	1.98	0.2	1.0	0.52	1.85	0.2	1.0
Tetrachloroethylene	0.27	2.21	0.2	1.3	0.27	2.25	0.2	1.6
Trichloroethylene	0.49	1.23	0.5	0.5	-	-	-	-
Toluene	13.4	2.07	4.4	45.6	13.6	2.04	4.3	52.2
*o*-Xylene	1.68	2.38	0.3	7.9	1.68	2.47	0.3	6.6
*p*-Xylene	5.77	1.88	1.7	17.4	6.08	1.96	1.9	17.9
Total xylenes	7.28	2.16	1.4	25.4	7.81	2.07	2.1	24.6
NO_2_	18.7	2.07	2.5	49.1	36.3	2.36	5.2	141

-: Calculation was not performed because all measured values were under the detection limit.

Table 4 shows a list of residents who had reported that they had experienced symptoms since their move into the flats. A total of seven males and 10 females had experienced symptoms in their bedrooms; three males and five females had experienced symptoms in their kitchens. Among them, two males and two females still experienced symptoms when in their bedrooms, and two females experienced symptoms when in their kitchens at the time of study. Table 5 presents the subjective symptoms reported by these residents (males of code 5 and 8 reported that they experienced some symptom, but did not specify it).

**Table 4:** A list of residents reported that they experienced some symptom because they moved into flats: before and during the sampling period.

	Code	Age (year)	Period living in this flat. (months)	Bedroom		Kitchen	
				Before	During	Before	During
*Males*							
	1	27	10	+	+		
	2	30	29	+		+	
	3	31	36	+			
	4	32	41	+			
	5	34	30	+	+	+	
	6	52	130	+			
	7	67	9	+			
	8	NA	NA			+	
*Females*							
	1	27	NA	+			
	2	30	29	+	+	+	+
	3	27	36	+			
	4	34	41	+			
	5	30	30	+	+	+	+
	6	52	NA	+		+	
	7	64	9	+			
	9	36	NA	+			
	10	51	19	+		+	
	11	NA	NA	+		+	

NA: No answer. +: Report of experience of symptom.

**Table 5:** Symptoms experienced after residents moved into their flats (shown by code in Table 3).

	Bedroom		Kitchen	
	Males (n = 59)	Females (n = 50)	Males (n = 59)	Females (n = 50)
Feeling of heavy-headedness		1, 7		
Headache	3	3, 5 ^a^		
Nausea	3, 7	3		
Nightmare		2 ^a^, 7		
Sleeplessness	6	1, 6		
Feel dizzy when stand up	7	1, 2 ^a^, 6		2 ^a^
Fatigue		1, 2 ^a^,5 ^a^, 10		2 ^a^
Slight fever		10		
Painful eye		9, 10		5 ^a^
Feeling of collapse		2 ^a^, 10		2 ^a^
Itchy eyes	4	4		
Nasal irritation	2	11	2	11
Whistles	6			
Sore throat				5 ^a^
Strange taste	1 ^a^, 2	9	2	5 ^a^
Itchy skin				10
Rash		5 ^a^		10
Muscle pain	1 ^a^			
Smell of paints and adhesives	4	4	5	

Blank: no subjects. Males of No. 5 and No. 8 did not specify symptoms they experienced. ^a^ Residents still had symptoms at the time of study (during sampling).

**Figure 1:**
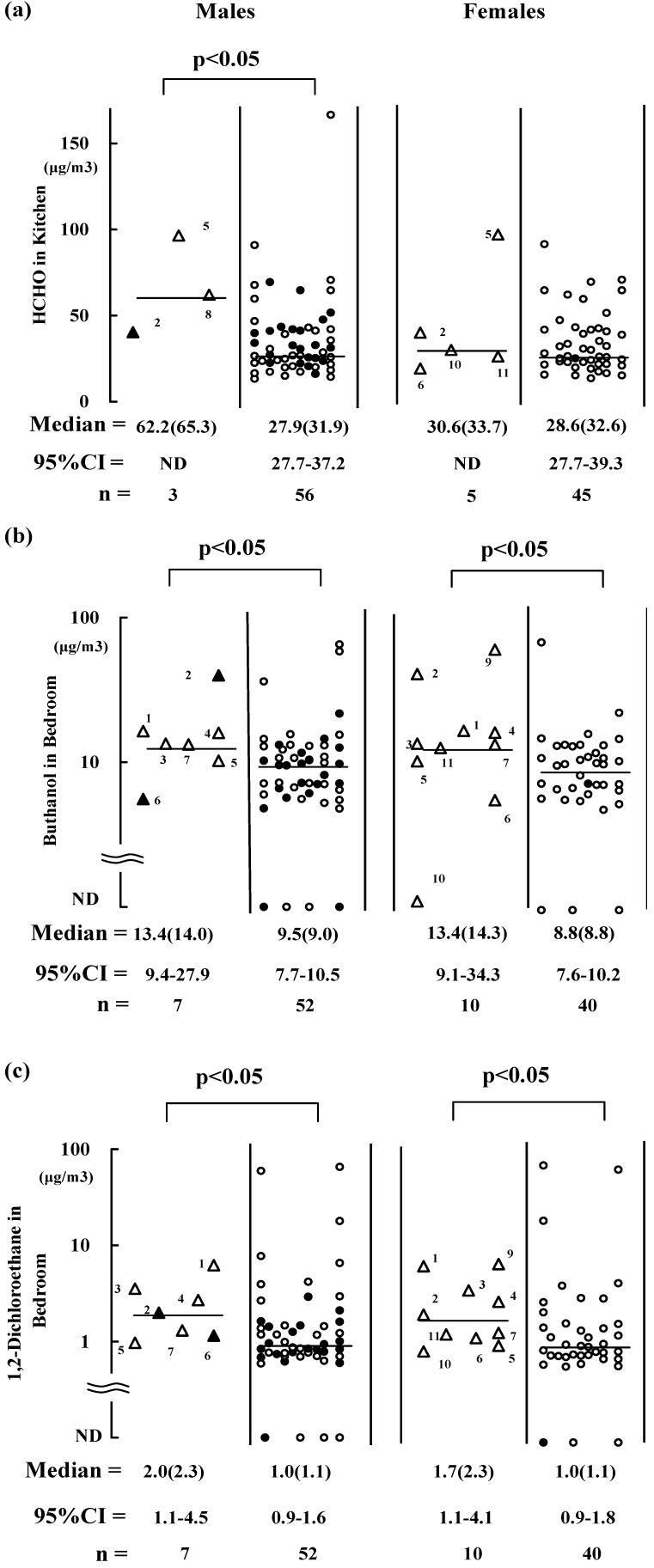
Air concentrations of HCHO, butanol, and 1, 2-dichloroethane in residences are shown in (**a**), (**b**), and (**c**), respectively. Residents with and without subjective symptoms after they moved into the flats are represented by (Δ,▲) and (○, ●), respectively (closed triangles and circles are smokers). Bar = median value. Point estimation of median is in parenthesis; 95% CI = 95% confidence interval. ND = not determined because of the small number of subjects.

**Figure 2:**
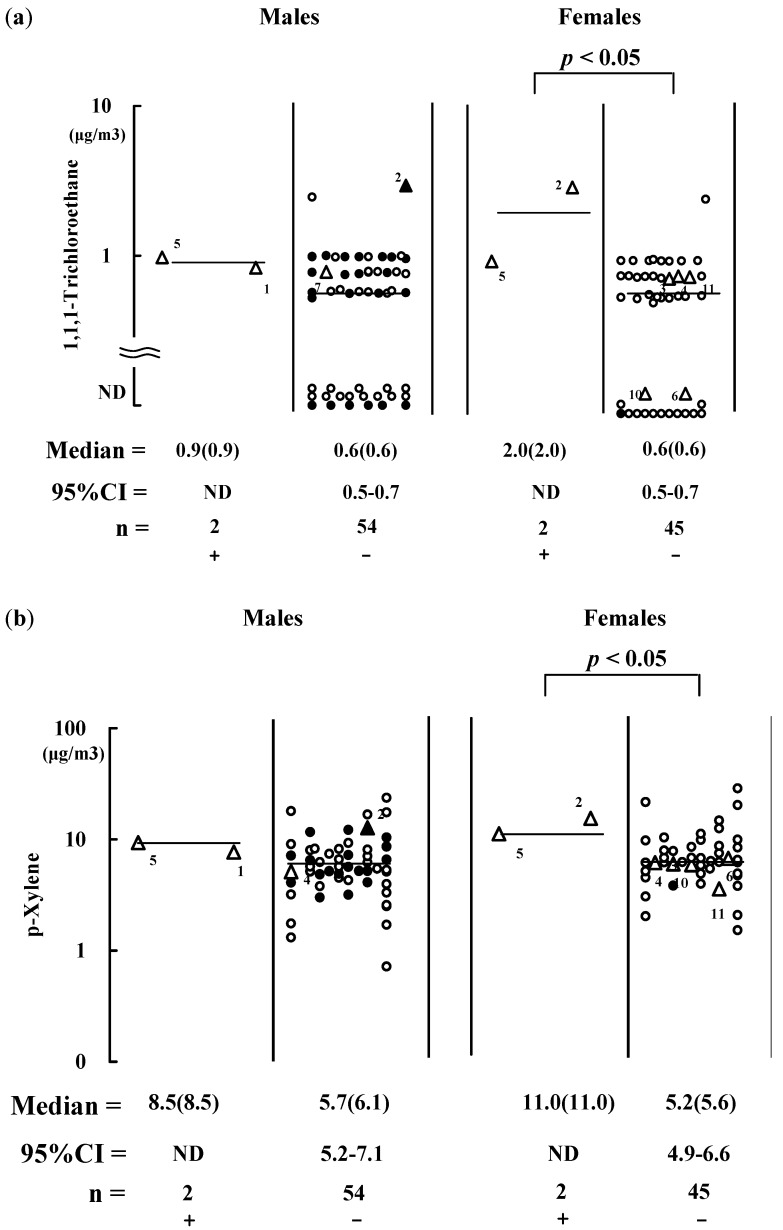
Air concentrations of 1,1,1-trichloro-ethane (**a**) and p-xylene (**b**) in bedrooms. These values were compared between residents with (+) and without (−) symptoms during the sampling. Symbols are specified in Figure 1. Bar = median value. Point estimation of median is in parenthesis; 95% CI = 95% confidence interval. ND = not determined because of the small number of subjects.

**Figure 3:**
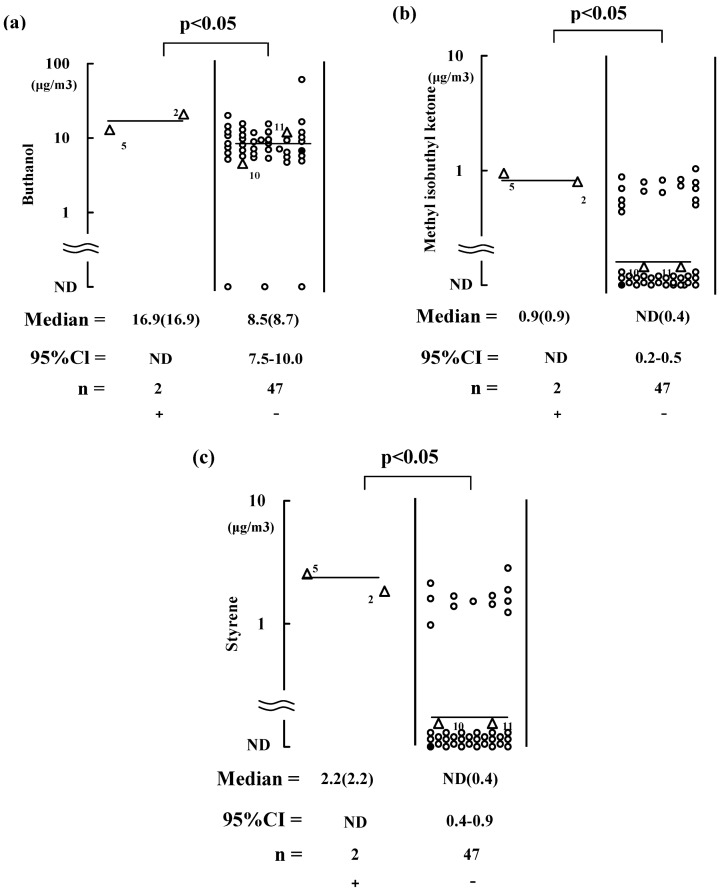
Air concentrations of butanol (**a**), methyl isobutyl ketone (**b**), and styrene (**c**) in kitchens. These values were compared between females with (+) and without (−) symptoms during the sampling. Symbols are specified in Figure 1. Bar = median value. Point estimation of median is in parenthesis; 95% CI = 95% confidence interval. ND = not determined because of the small number of subjects.

Figure 1, Figure 2, Figure 3 are presented to show the significant differences observed in indoor concentrations of chemicals between residents with and without symptoms before the sampling after they had moved into their flats or during the sampling. Residents with and without symptoms are indicated by triangles and circles, respectively (smokers are indicated as closed one). Three males experienced symptoms before the sampling in the kitchens where the median HCHO level was 62.2 μg/m^3^ (Figure 1(a)). Additionally, five females had symptoms before the sampling in the kitchens, although the present HCHO levels were not significantly elevated (Figure 1(a)). No significant differences in indoor HCHO levels of the residents with symptoms were found compared with those without symptoms during the sampling (*p* > 0.05, data not shown). Therefore, it has been suggested that HCHO levels before the sampling were greater than 60.0 μg/m^3^, which has been reported to cause symptoms related to nervous or respiratory systems [10] and that HCHO concentrations decreased with the aging of the sampled residences [14].

Seven males and 10 females reported that they felt at least one of the symptoms when they were in their bedrooms before the sampling (Figure 1(b,c)). The median butanol concentration in their bedrooms was 13.4 μg/m^3^ for both males and females (Figure 1(b)). This value was greater than the median butanol concentrations in the bedrooms in which the residents did not report any symptoms, *i.e.*, 9.53 μg/m^3^ and 8.84 μg/m^3^ for males and females, respectively (Figure 1(b)). Among these 10 females, two (code 2 and 5) still had symptoms during the sampling in the kitchens where the median butanol level was 16.9 μg/m^3^ (Figure 3(a)). Therefore, relatively high-level exposure to butanol (*i.e*., median 13–16.9 μg/m^3^) might have caused the symptoms. Median values of 1,2-dichloroethane in the bedrooms (2.0 and 1.7 μg/m^3^ for males and females, respectively) were significantly greater for those with symptoms before the sampling compared with those without symptoms (1.0 μg/m^3^ ) (Figure 1(c)), whereas a significantly elevated concentration of 1,2-dichloroethane was not observed for residents with symptoms during the sampling. It is also assumed that the concentration of 1,2-dichloroethane decreased with the aging of the sampled residences.

The two females (code 2 and 5) also had symptoms during the sampling related to elevated levels of 1,1,1-trichloroethane and p-xylene in the bedrooms, *i.e.*, at median levels of 2.00 and 11.0 μg/m^3^, respectively (Figure 2(a,b)). Two males (one was the husband of female code 5) complained of symptoms in the bedrooms, but their exposure to 1,1,1-trichloroethane and p-xylene levels were not significantly elevated (Figure 2(a,b)). This might have been because the husband of female of code 2 did not manifest the symptom. The females (code 2 and 5) still had symptoms related to methyl isobutyl ketone and styrene in the kitchens, with median levels of 0.90 and 2.20 μg/m^3^, respectively (Figure 3(b,c)). Therefore, concentrations of butanol, 1,1,1-trichloroethane, *p-*xylene, methyl isobutyl ketone and styrene were elevated for subjects with persisting symptoms most likely because these chemical concentrations did not rapidly decrease.

The subjective symptoms observed in the present study could be attributed to indoor chemicals. However, most of these chemical substances were detected at very low concentrations. The analysis was performed under a total-ion monitoring mode for some samples to detect all of the major peaks, followed by selected-ion monitoring mode to target the quantified VOCs, which did not reveal high levels of VOCs other than those reported here. To confirm that such low exposures can cause long-term adverse effects, further study is necessary. Several studies have reported that total VOC concentration was significantly related to symptoms of SBS [2,20,21]. However, in the present study, no significant differences were observed in airborne total VOC between the rooms of participants with and without symptoms. Reasons for this discrepancy should be further studied. Additionally, short but higher-level exposure should be considered even though the measured average exposure level was low. In addition, decreases in air concentrations might have been caused by opened windows during the sampling.

Dampness is known to be associated with respiratory tract infections in dwellings [22,23,24] and office buildings [25]. It has been reported that environmental tobacco smoke [26] is a risk for SBS symptoms. The effects of indoor dampness and environmental tobacco smoke on the health of residents should have been evaluated in the present study. The effects of indoor chemical substances on children should also be evaluated because it has been reported that childhood asthma is related to formaldehyde [27,28,29] and VOC exposure [30].

## 4. Conclusions

Although the current indoor exposure to HCHO, butanol, or 1,2-dichloroethane seems very low, previous high-level exposure to these chemicals should be considered among residents who once experienced symptoms related to SBS. Additionally, these symptoms could be caused by current low-level exposure to butanol, 1,1,1-trichloroethane, *p*-xylene, methyl isobutyl ketone or styrene. Exposure limits of these chemicals should be evaluated and established by further study to prevent SBS.

## Appendix

**Appendix 1:** Number (%) of measured values below detection limits.

	Bedroom	Kitchen	Outdoor
Formaldehyde (HCHO)	0 (0)	0 (0)	0 (0)
VOC (total)	0 (0)	0 (0)	0 (0)
Benzene (C_6_H_6_)	1 (1.69)	1 (1.69)	2 (5.13)
Butanol (C_4_H_10_O)	6 (10.2)	3 (5.08)	16 (41.03)
Butyl acetate (C_6_H_12_O_2_)	2 (3.39)	2 (3.39)	5 (12.8)
Carbon tetrachloride (CCl_4_)	16 (27.1)	13 (22.03)	10 (25.6)
Chloroform (CHC_l3_)	54 (91.5)	56 (94.9)	38 (97.4)
1,2-Dichloroethane (C_2_H_4_C_l2_)	4 (6.78)	2 (3.39)	6 (15.4)
*p*-Dichlorobenzene (C_6_ H_4_C_l2_)	28 (47.5)	30 (50.9)	34 (97.2)
Ethyl acetate (C_4_H_8_O_2_)	59 (100)	59 (100)	39 (100)
Hexane (C_6_H_14_)	3 (5.08)	3 (5.08)	1 (2.56)
Methyl ethyl ketone (CH_3_COC_2_H_5_)	45 (76.3)	47 (79.7)	31 (79.5)
Methyl isobutyl ketone (CH_3_COCH_2_CH(CH_3_)_2_)	33 (55.9)	37 (62.7)	31 (79.5)
Styrene (C_6_H_5_C_2_H_3_)	58 (98.3)	56 (94.9)	33 (94.6)
1,1,1-Trichloroethane (CH_3_CCl_3_)	21 (33.6)	19 (32.2)	16 (41.03)
Tetrachloroethylene (C_2_Cl_4_)	57 (96.6)	56 (94.9)	36 (92.3)
Trichloroethylene (C_2_HCl_3_)	57 (96.6)	59 (100)	39 (100)
Toluene (C_6_H_5_CH_3_)	0 (0)	0 (0)	0 (0)
*o*-Xylene (C_8_H_10_)	4 (6.78)	5 (8.47)	9 (23.08)
*p*-Xylene (C_8_H_10_)	0 (0)	0 (0)	1 (2.56)
Total xylenes (C_8_H_10_)	2 (3.39)	0 (0)	4 (10.3)
NO_2_	0(0)	0 (0)	0 (0)

**Appendix 2:** Point estimation and 95% confidence intervals (CI) of median values in Table 2.

	Bedroom		Kitchen		Outdoor	
	Point estimation	95%CI	Point estimation	95%CI	Point estimation	95%CI
Formaldehyde	34.4	28.9–40.1	33.1	28.7–39.8	15.6	13.1–17.9
VOCs (total)	49.2	41.2–61.5	49.3	42.5–58.2	33.9	28.2–40.2
Benzene	2.56	2.26–2.93	2.91	2.60–3.34	2.49	2.16–2.91
Butanol	9.72	8.37–11.3	9.02	7.88–10.4	5.21	4.31–6.32
Butyl acetate	3.83	3.25–4.69	3.72	3.24–4.28	2.95	2.37–3.67
Carbon tetrachloride	0.78	0.68–0.91	0.81	0.73–0.95	0.81	0.64–0.97
Chloroform	0.82	0.82–0.82	0.82	0.82–0.82	0.82	0.82–0.82
1,2-Dichloroethane	1.22	1.00–1.80	1.21	1.04–1.71	1.01	0.83–1.19
*p*-Dichlorobenzene	0.39	0.26–0.44	0.39	0.26–0.46	0.26	0.26–0.26
Ethyl acetate	20.3	20.3–20.3	20.3	20.3–20.3	20.3	20.3–20.3
Hexane	1.6	1.38–1.84	1.7	1.48–1.94	1.67	1.36–1.99
Methyl ethyl ketone	0.62	0.62–0.97	0.62	0.62–1.02	0.62	0.62–1.09
Methyl isobutyl ketone	0.42	0.31–0.48	0.38	0.21–0.48	0.21	0.21–0.36
Styrene	0.39	0.39–0.89	0.68	0.39–0.96	0.39	0.39–0.96
1,1,1-Trichloroethane	0.60	0.50–0.68	0.61	0.50–0.68	0.50	0.41–0.67
Tetrachloroethylene	0.20	0.20–0.20	0.20	0.20–0.20	0.20	0.20–0.20
Trichloroethylene	0.47	0.47–0.47	0.47	0.47–0.47	0.47	0.47–0.47
Toluene	14.8	12.1–18.1	14.5	11.9–17.9	10.3	8.79–12.4
*o*-Xylene	1.95	1.58–2.39	1.90	1.58–2.30	1.50	1.11–1.93
*p*-Xylene	6.20	5.39–7.19	6.30	5.55–7.16	5.47	4.40–6.76
Total xylenes	8.25	7.00–9.67	8.24	7.12–9.49	6.94	5.40–8.69
NO_2_	22	18.6–25.8	44	36.4–51.8	31.2	25.0–38.0
